# Self-reported use of family physician, chiropractor and physiotherapy services among adult Canadians with chronic back disorders: an observational study

**DOI:** 10.1186/s12913-018-3790-6

**Published:** 2018-12-17

**Authors:** Brenna Bath, Josh Lawson, Dennis Ma, Catherine Trask

**Affiliations:** 1 School of Rehabilitation Science & Canadian Centre for Health and Safety in Agriculture (CCHSA), College of Medicine, University of Saskatchewan, Health Sciences Building, E-Wing, Suite 3400, 104 Clinic Place, Saskatoon, S7N 2Z4 SK Canada; 2 Canadian Centre for Health and Safety in Agriculture (CCHSA), University of Saskatchewan, 104 Clinic Place, Saskatoon, S7N 2Z4 SK Canada; 3 Sauder School of Business, University of British Columbia, Vancouver, Canada

**Keywords:** Low back pain, Health services accessibility, Physician services, Physical therapy, Chiropractic

## Abstract

**Background:**

Chronic back disorders (CBD) are prevalent, costly, and among the most common reasons for seeking primary care; however, little is known regarding the comparative use of family physician, chiropractic, and physiotherapy services among people with CBD in Canada. Elucidating these differences may identify potential gaps in access to care and inform the development of strategies to improve access. The research objectives were to investigate patterns of health care use and to profile factors associated with self-reported use of family physicians, chiropractors, and physiotherapists among adult Canadians with CBD.

**Methods:**

The combined 2009 and 2010 Canadian Community Health Surveys conducted by Statistics Canada were used to investigate self-reported health care use among adults with CBD. This complex survey employs population weights and bootstrapping to be representative of the Canadian population. Following descriptive analyses, we used multiple logistic regression to profile self-reported health care use while statistically controlling for possible confounding effects.

**Results:**

The majority of adult respondents with CBD sought care only with a family physician (53.8%), with 20.9% and 16.2% seeking care with combined family physician/chiropractor or family physician/physiotherapist, respectively. Few respondents sought care only with a chiropractor (2.5%) or physiotherapist (1.0%). After adjustment, differential patterns of utilization (*p* < 0.05) were evident between provider groups with respect to age, gender, socioeconomic status, rural/urban residence, functional limitations, and presence of co-morbidities.

**Conclusions:**

This research highlights potential inequities in access to physiotherapists and chiropractors in relation to family physicians among adult Canadians with CBD, particularly among lower socioeconomic status and rural/remote populations.

## Background

Chronic back disorders (CBD) are a prevalent and costly public health issue; however, little is known about the patterns of community-based health care use among Canadians with these common and potentially disabling conditions. Compared to 289 other diseases and conditions, low back pain is the leading cause of morbidity worldwide when considering years lived with disability [1]. In Canada, 22% of adults report having back problems lasting 6 months or more [2] and health care expenditures are estimated between $6 and $12 billion annually [3]. Back disorders are costly to individuals and strain health care resources due to high rates of primary physician care visits, [4, 5] specialist consultations, diagnostic procedures [6, 7], and prescribed medications such as opioids [8]. Limited access to appropriate primary care is thought to be a contributing factor to this “medical disaster” [9].

Although family physicians are typically the first clinical contact for people with low back disorders [5], they may not be the most appropriate health care provider to assess and treat these conditions [10, 11]. Conversely, physiotherapists have been shown to have high levels of competency in the assessment, diagnosis, and management of low back disorders and are extensively trained in non-pharmacological pain management approaches [12, 13]. Furthermore, adding clinical services that physiotherapists and chiropractors are trained to offer (such as exercise prescription and manual therapy) to usual general medical practitioner care for low back disorders are more cost-effective than usual medical care alone [14]. Unfortunately, access to community-based (i.e. outside of hospitals) or privately delivered physiotherapy and chiropractor services may be limited to those who have additional health insurance or are able to pay, with investigation and treatment for people with CBD who cannot afford these services falling, often inappropriately, to publicly-funded medical care [15–17]. Although people in Canada can access physiotherapy and chiropractic services directly without a physician referral, several barriers may result in those with CBD seeking care first (or only) with a family physician whose care is fully funded by the public system. For example, lack of awareness of how or when to access these non-physician services, as well as the misconception that a physician referral is required to receive reimbursement from insurance companies outside of the publicly funded system.

Despite back disorders being the most common pain problem in the general population [18] and one of the most common reasons for seeking health care [5, 15]; little is known regarding the comparative use of family physician, chiropractic, and physiotherapy services in Canada. Elucidating these differences may help to identify potential gaps in access to care and may assist in the development of strategies to optimize equitable access. The objectives of this research were to: 1) investigate patterns of use of family physician, chiropractor, and physiotherapy services; and 2) profile the sociodemographic and other factors associated with use of different health care providers among adult Canadians with CBD.

## Methods

### Study design and data source

We used combined data from Statistics Canada’s 2009 and 2010 Canadian Community Health Surveys (CCHS). The CCHS was designed to provide a flexible, broad based survey instrument to address emerging health issues in Canada. It includes a range of content, including: socio-demographics; health status; health behaviours; and many other determinants of health [19]. The CCHS is a cross-sectional survey in which respondents are selected using a complex survey design with a two-phase stratified sampling plan intended to ensure adequate representation from each Canadian region.

### Study population

The CCHS targets Canadians 12 years and older living in private dwellings in all 10 provinces and 3 territories. The survey did not include people living on First Nations reserves or residents of institutional and some non-institutional collectives (e.g., military bases, Canadian Armed Forces vessels, merchant and coast guard vessels, campgrounds or parks). Approximately 130,000 Canadians were selected for the 2009 or 2010 survey, sampled from and representative of approximately 98% of the Canadian population aged 12 years and older. The participation rate of this survey was 72.3% [19]. The focus of our analysis was persons aged 18 years and older who had not been hospitalized in the past year (*N* = 113,647). People who were hospitalized in the past year were removed from the analysis in order to focus on ‘community-based’ service provision and to eliminate respondents who may have received care from one of the providers of interest within an acute care setting (i.e. hospital). Of these adult respondents, 25,545 reported having CBD.

### Survey data and operational definitions

The presence of CBD was identified, using the survey question: “(Do you) have back problems, excluding fibromyalgia and arthritis?” This section of the survey is prefaced with the reminder: “Now I’d like to ask about certain chronic health conditions which (you) may have. We are interested in ‘long-term conditions’ which are expected to last or have already lasted 6 months or more and that have been diagnosed be a health professional.”

The dependent variables were self-reported use of the following health care providers in the past 12 months: family physicians, chiropractors, or physiotherapists. Family physician use was further refined to include ‘only’ use by a family physician (i.e. no use of a chiropractor or physiotherapist), indicating use of predominantly publically funded services. Due to the relatively small sample of respondents who reported ‘only’ use of either chiropractic or physiotherapy services, we considered ‘any’ use of these health care providers in the analysis. This resulted in the following three dichotomized (i.e. yes/no) variable groups: 1)only family physician use; 2) any chiropractor use; and 3) any physiotherapy use. Because of the definitions used, the latter two groups are not mutually exclusive. A range of independent variables grouped into sociodemographic, lifestyle, and health characteristics were identified based on a review of the literature, clinical relevance, and data availability within the survey. Further details regarding the description and categorization of the variables can be found in Table 1.

**Table 1:** Description of independent variables included in analysis

Variable	Description (if applicable) & categories
Health care utilization	
Family Physician	Self-reported use obtained from the question: “(In the past 12 months) Have you seen or talked to a family doctor or general practitioner about your physical, emotional or mental health?”. This included respondents with “only” use of a family physician (i.e. no use of a chiropractor or physiotherapist)
Chiropractor	Self-reported use obtained from the question: “(In the past 12 months) Have you seen or talked to a chiropractor about your physical, emotional or mental health?” This included respondents with “any” use of a family chiropractor (i.e. could also report use of a family physician or physiotherapist).
Physiotherapist	Self-reported use obtained from the question: “(In the past 12 months) Have you seen or talked to a physiotherapist about your physical, emotional or mental health?” This included respondents with “any” use of a physiotherapist (i.e. could also report use of a family physician or chiropractor).
Socio-demographic characteristics	
Age	18–34 yrs.; 35-49 yrs.; 50-64 yrs.; ≥ 65 yrs. Categories based on quartiles and clinical relevance
Sex	Male; female
Education	Less than secondary; secondary graduation; some post-secondary; post-secondary graduation
Income	A StatsCan-derived variable addressing income adequacy. Quintile of adjusted ratio of total household income to the low income cut-off corresponding to household and community size. This variable was unavailable for some respondents, for example, in cases where the person most knowledgeable about the household could not be identified.
Residence	A StatsCan-derived variable. “Urban” residence includes communities with populations ≥10,000 people. “Rural” communities are disaggregated into sub-groups or Metropolitan Influenced Zones (MIZ) based on the size of commuting flows to any larger urban centre [41].
Ethnicity	Caucasian; Aboriginal (i.e. First Nation, Métis or Inuit); other
Marital status	Single; married or common law; widowed or separated or divorced
Immigration status	Canadian-born; immigrant
Body Mass Index (BMI)	Derived from self-reported height and weight. Underweight & Normal (< 25 kg/m^2^); Overweight (25–29.9 kg/m^2^); Obese (≥ 30 kg/m^2^) [42]
Lifestyle characteristics	
Smoking status	Never smoked; former smoker; current smoker
Physical activity	A StatsCan derived variable combining leisure time and transportation physical activity based on estimated total daily energy expenditure variables (kcal/kg/day): active; moderately active; inactive
Health characteristics	
Number of other co-morbidities/chronic conditions	Includes “long-term conditions” which are expected to last or have already lasted 6 months or more and that have been diagnosed by a health professional. No other chronic conditions (other than CBD); 1or 2 chronic conditions (other than CBD); 3 or more chronic conditions (other than CBD)
HUI Pain/Function	Health Utility Index (HUI) variable [43]. Considers whether pain prevents person from performing activities of daily living. 5 categories: no pain or discomfort; pain prevents no activities; pain prevents a few activities; pain prevents some activities; pain prevents most activities
Self-rated stress	Ability to handle day-to-day demands: not at all/not very; a bit; quite a bit/extremely*
Self-rated overall health	Indicates the respondent’s health status based on his/her own judgement or his/her proxy: excellent/very good; good; fair/poor^a^

^a^Collapsing of these categories was performed to maintain equal-sized categories and consistent categorization for all variables of interest

### Statistical analysis

Patterns of health care use were determined by calculating proportions for use of each health care provider group among adults with CBD. The descriptive analysis of factors associated with health care use included calculation of proportions over each of our independent variables for persons who report seeking care with each health care provider. Base probabilities between each independent variable and each health care provider use type were assessed using bivariate logistic regression. The strength of association was estimated using odds ratios (OR) with a 95% confidence interval (CI).

Any variable for which the bivariate analysis produced a *p*-value < 0.25 was considered a candidate for the multivariate analysis. We then applied Spearman’s correlation coefficient (*r*) to evaluate the association between independent variables; among variables that were correlated (*r* > 0.5), we included only the most significant in the multivariate analysis, to avoid multicollinearity in the multivariate model. Backwards stepwise selection (logistic regression) was used to select from the remaining independent variables with *p*-values of 0.10 or larger to exit model and 0.05 or lower to enter [20, 21]. Specifically, we employed a recursive four step approach. First, the independent variable with the highest *p*-value was identified from the set of independent variables. Second, the identified variable was removed from the set and added to the set of removed variables conditional on passing the exit threshold. Third, the set of removed variables, except for the latest, were included in a regression on top of the current set of independent variables. Fourth, the previously removed variables were re-evaluated, and any variables with *p*-values less than the entry threshold were moved back into the independent variable set. The process ends when no more independent variables fall below the exit threshold. Categorical variables were evaluated based on the lowest p-value of the category set, and not as individual dummies. A stepwise selection procedure is recommended for exploratory procedures analyzing the relationships among dependent outcome variables that are not established, and backwards stepwise selections reduce the risk of Type II error [21].

All final multivariate models were adjusted for province of residence to account for variations in provincial health funding policies and health care provider distribution.

All analyses were performed using Stata® 13 software with built-in survey data tools for probability weights and bootstrapping. Probability weights provided by Statistics Canada were used and, in order to account for the complex survey design, bootstrap methods for robust variance estimation were employed using provided bootstrap weights to accurately estimate standard errors.

## Results

**Fig. 1:**
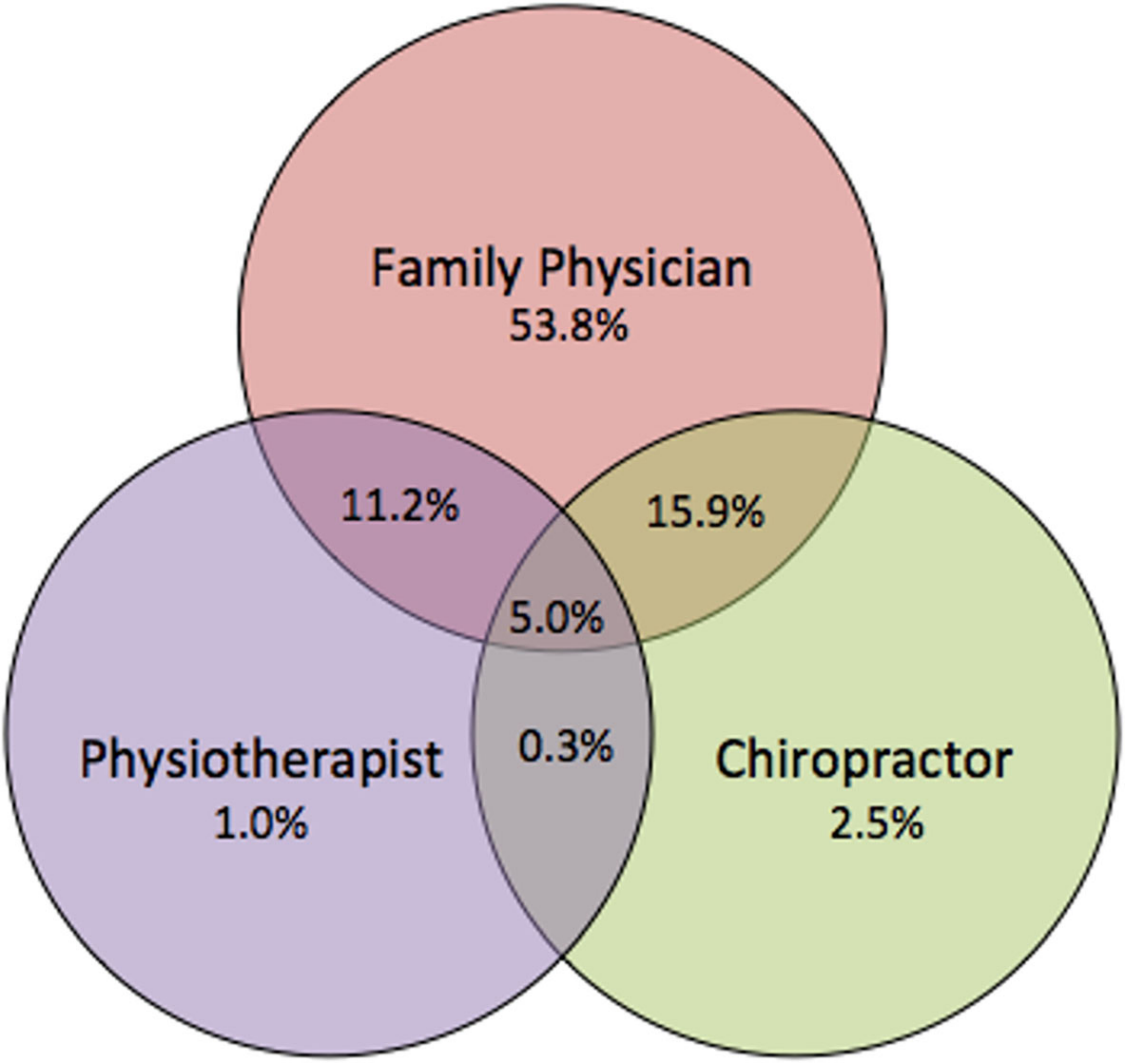
Prevalence of self-reported health care use among adult Canadians with CBD

The prevalence of self-reported use of each of the health care provider groups can be found in Fig. 1. The majority of respondents with CBD sought care only with a family physician (53.8%), with only 2.5 and 1% seeking care only with a chiropractor or physiotherapist respectively. Respondents reported seeking care with the following combination of providers: chiropractor/family physician (20.9%); physiotherapist/family physician (16.2%); physiotherapist/chiropractor (0.3%); family physician/chiropractor/physiotherapist (5.0%).

The results of the descriptive analysis for each health care provider group are presented in Table 2. Due to Statistics Canada’s strict confidentiality and vetting guidelines, we were unable to report certain low prevalence values (i.e. ethnicity other than Caucasian, strongly and moderately influenced MIZ among physiotherapist use group).

**Table 2:** Socio-demographic, Lifestyle and Health Characteristics among Canadians with CBD by health care provider groups

Respondent characteristic	Family physician only^a^ (%)	Chiropractor any^b^ (%)	Physiotherapist any^b^ (%)
Age			
18-34	13.24	19.99	18.50
35–49	25.31	32.97	32.22
50–64	34.19	33.96	34.42
65+	27.26	13.09	14.86
Sex- male	45.12	47.81	44.63
Education			
- less than secondary	22.52	11.53	11.56
- secondary graduation	16.40	16.96	14.13
- some post-secondary	7.50	7.52	6.09
- post-secondary graduation	53.59	63.99	68.21
Income Quintile			
- 1	28.24	12.22	15.48
- 2	21.84	19.65	19.63
- 3	18.17	21.65	18.26
- 4	15.76	22.09	20.00
- 5	16.00	24.39	26.62
Residence			
- urban/metropolitan	70.81	70.88	77.16
- strongly influenced MIZ	3.72	3.92	^c^
- moderately influenced MIZ	7.57	7.95	^c^
- weak/uninfluenced MIZ & territories	17.90	17.26	14.41
Ethnicity			
- caucasian	83.36	87.37	81.69
- aboriginal	^c^	^c^	^c^
- other	^c^	^c^	^c^
Marital status			
- Single	15.05	17.01	16.88
- Married+Common-law	64.63	70.47	68.48
-Widowed+Seperated+Divorced	20.32	12.52	14.63
Immigration Status- Canada born	76.00	81.29	77.17
immigrant	24.01	18.71	22.83
Smoking Status			
- never smoked	30.91	34.70	33.02
- former smoker	41.79	44.29	46.03
- current smoker	27.30	21.01	20.95
BMI			
- underweight/normal	39.05	40.46	42.37
- overweight	36.68	38.58	37.61
- obese	24.27	20.96	20.01
No. of co-morbidities			
- none	23.37	34.90	28.71
- 1-2	47.19	48.98	49.89
- 3+	29.43	16.13	21.40
Physical Activity			
- inactive	56.88	46.96	48.81
- moderately active	22.64	27.10	24.18
- active	20.48	25.94	27.07
HUI Pain/Function			
- no pain or discomfort	55.02	55.41	43.06
- pain prevents no activities	9.46	10.15	8.94
- pain prevents a few activities	13.39	16.61	18.52
- pain prevents some activities	12.69	11.31	16.97
- pain prevents most activities	9.44	6.52	12.52
Self-rated Stress			
- not at all/not very	29.78	25.72	26.02
- a bit	40.11	43.77	38.71
- quite a bit/extremely	30.11	30.51	35.27
Self-Rated Overall Health			
- excellent/very good	38.48	51.15	43.08
- good	34.46	34.35	34.65
- fair/poor	27.06	14.50	22.27

Abbreviations: *MIZ* metropolitan influenced zone; *BMI* body mass index; *HUI* health utility index

^a^use of only family physician services (i.e. no reported use of chiropractor or physiotherapist services)

^b^any use of family physician, chiropractor and/or physiotherapist services

^c^unable to report due to Statistics Canada’s confidentiality and vetting requirements

Table 3 presents our multivariate models for each health care provider group along with the unadjusted (bivariate) logistic regression results for each variable included in the model. Bolded values were statistically significant at *p* < 0.05 level.

**Table 3:** Association of health care utilization with socio-demographic, lifestyle and health characteristics among respondents with back disorders^a^

Respondent Characteristic	Family Physician (only)				Chiropractor (any)				Physiotherapist (any)			
	Unadjusted		Adjusted		Unadjusted		Adjusted		Unadjusted		Adjusted	
	OR	95%CI	OR	95%CI	OR	95%CI	OR	95%CI	OR	95%CI	OR	95%CI
Age												
18–34 (ref)	1.00	–	1.00	–	–	**–**	–	**–**	1.00	**–**	1.00	**–**
35–49	**1.21**	**1.02–1.43**	**1.22**	**1.02–1.45**	**–**	**–**	**–**	**–**	0.99	0.78–1.24	0.84	0.66–1.07
50–64	**1.56**	**1.33–1.83**	**1.42**	**1.19–1.69**	**–**	**–**	**–**	**–**	0.88	0.69–1.11	**0.76**	**0.60–0.98**
65+	**3.05**	**2.62–3.56**	**2.18**	**1.81–2.62**	**–**	**–**	**–**	**–**	**0.58**	**0.47–0.73**	**0.59**	**0.45–0.75**
Sex												
Male (female ref)	**0.76**	**0.69–0.84**	**0.84**	**0.75–0.93**	**–**	**–**	**–**	**–**	**0.79**	**0.68–0.92**	**0.78**	**0.67–0.91**
Education												
- less than secondary (ref)	1.00	–	1.00	–	1.00	**–**	1.00	**–**	1.00	**–**	1.00	**–**
- secondary graduation	**0.61**	**0.51–0.73**	0.87	0.72–1.04	**2.04**	**1.56–2.59**	**1.48**	**1.17–1.89**	**1.43**	**1.07–1.91**	1.14	0.85–1.54
- some post-secondary	**0.66**	**0.53–0.83**	0.93	0.74–1.18	**1.82**	**1.37–2.41**	**1.40**	**1.06–1.87**	1.37	0.97–1.92	1.03	0.73–1.44
- post-secondary graduation	**0.52**	**0.45–0.61**	**0.82**	**0.71–0.95**	**2.05**	**1.68–2.50**	**1.41**	**1.15–1.72**	**2.06**	**1.68–2.53**	**1.49**	**1.19–1.86**
Income Quintile												
- 1 (lowest- ref)	1.00	–	1.00	–	1.00	**–**	1.00	**–**	1.00	**–**	1.00	**–**
- 2	**0.63**	**0.54–0.74**	**0.72**	**0.61–0.84**	**1.89**	**1.53–2.33**	**1.64**	**1.32–2.03**	**1.44**	**1.12–1.84**	**1.50**	**1.16–1.94**
- 3	**0.52**	**0.45–0.62**	**0.67**	**0.57–0.79**	**2.50**	**2.05–3.07**	**1.99**	**1.61–2.46**	**1.43**	**1.14–1.80**	**1.57**	**1.24–1.99**
- 4	**0.47**	**0.40–0.55**	**0.68**	**0.57–0.82**	**2.87**	**2.36–3.50**	**2.10**	**1.70–2.59**	**1.82**	**1.45–2.28**	**1.91**	**1.51–2.40**
- 5 (highest)	**0.40**	**0.34–0.47**	**0.59**	**0.49–0.71**	**2.91**	**2.39–3.54**	**1.97**	**1.60–2.43**	**2.33**	**1.85–2.93**	**2.40**	**1.86–3.10**
Residence												
- CMA or CA (urban) (ref)	**–**	**–**	**–**	**–**	**–**	**–**	**–**	**–**	1.00	–	1.00	–
- strongly influenced MIZ	**–**	**–**	**–**	**–**	**–**	**–**	**–**	**–**	**0.60**	**0.45–0.78**	**0.62**	**0.46–0.83**
- moderately influenced MIZ	**–**	**–**	**–**	**–**	**–**	**–**	**–**	**–**	**0.64**	**0.49–0.83**	**0.71**	**0.54–0.93**
- weak, uninfluenced MIZ & territories	**–**	**–**	**–**	**–**	**–**	**–**	**–**	**–**	**0.72**	**0.61–0.85**	**0.72**	**0.60–0.86**
Ethnicity												
- Caucasian (ref)	**–**	**–**	**–**	**–**	1.00	–	1.00	–	1.00	**–**	1.00	**–**
- Aboriginal	**–**	**–**	**–**	**–**	**0.70**	**0.53–0.93**	0.76	0.57–1.02	1.05	0.76–1.44	1.09	0.77–1.52
- Other	**–**	**–**	**–**	**–**	**0.68**	**0.51–0.90**	**0.69**	**0.52–0.90**	1.28	0.96–1.71	**1.41**	**1.04–1.92**
Immigrant												
(Canadian-born ref)	**1.29**	**1.11–1.50**	**1.28**	**1.09–1.49**	**–**	**–**	**–**	**–**	**–**	**–**	**–**	**–**
Smoking Status												
- never smoked (ref)	1.00	–	1.00	–	1.00	**–**	1.00	**–**	1.00	**–**	1.00	**–**
- former smoker	1.06	0.93–1.21	1.03	0.89–1.19	0.87	0.74–1.00	**0.84**	**0.72–0.97**	1.00	0.84–1.17	1.12	0.95–1.34
- current smoker	**1.21**	**1.04–1.41**	**1.25**	**1.06–1.46**	**0.63**	**0.52–0.75**	**0.68**	**0.57–0.82**	**0.67**	**0.54–0.84**	**0.76**	**0.60–0.95**
BMI												
underweight/normal (ref)	**–**	**–**	**–**	**–**	**–**	**–**	**–**	**–**	1.00	–	1.00	–
- overweight	**–**	**–**	**–**	**–**	–	–	–	–	0.95	0.80–1.14	0.97	0.81–1.17
- obese	**–**	**–**	**–**	**–**	–	–	–	–	**0.80**	**0.67–0.96**	0.82	0.68–1.00
No. of co-morbidities												
- none (ref)	1.00	–	1.00	–	1.00	**–**	1.00	**–**	**–**	**–**	**–**	**–**
- 1-2	**1.46**	**1.29–1.66**	**1.27**	**1.10–1.45**	0.84	0.73**–**0.98	0.87	0.75–1.00	**–**	**–**	**–**	**–**
- 3+	**2.88**	**2.48–3.34**	**1.89**	**1.57–2.28**	**0.51**	**0.42–0.61**	**0.64**	**0.52–0.78**	**–**	**–**	**–**	**–**
Physical activity												
- inactive (ref)	**–**	**–**	**–**	**–**	1.00	–	1.00	–	1.00	**–**	1.00	**–**
- moderately active	**–**	**–**	**–**	**–**	**1.38**	**1.20–1.60**	1.16	1.00–1.35	1.12	0.94–1.35	1.08	0.90–1.30
- active	**–**	**–**	**–**	**–**	**1.37**	**1.16–1.61**	1.07	0.91–1.26	**1.37**	**1.13–1.66**	**1.29**	**1.05–1.58**
HUI Pain/Function												
- no pain or discomfort (ref)	1.00	–	1.00	–	1.00	**–**	1.00	**–**	1.00	**–**	1.00	**–**
- pain prevents no activities	0.93	0.78–1.11	**0.77**	**0.64–0.92**	1.10	0.89–1.35	**1.29**	**1.03–1.60**	1.29	0.99–1.68	**1.46**	**1.12–1.90**
- pain prevents a few activities	0.94	0.81–1.10	**0.71**	**0.60–0.82**	**1.29**	**1.07–1.57**	**1.64**	**1.35–2.00**	**1.92**	**1.58–2.34**	**2.15**	**1.75–2.63**
- pain prevents some activities	1.12	0.95–1.32	**0.73**	**0.61–0.88**	0.87	0.72–1.06	1.18	0.95–1.46	**2.11**	**1.70–2.62**	**2.57**	**2.05–3.22**
- pain prevents most activities	**1.30**	**1.05–1.60**	**0.73**	**0.61–0.88**	**0.66**	**0.52–0.85**	1.10	0.83–1.45	**2.30**	**1.75–3.00**	**3.56**	**2.70–4.69**
Self-rated Stress												
- not at all/not very (ref)	1.00	–	1.00	–	1.00	**–**	1.00	**–**	**–**	**–**	**–**	**–**
- a bit	**0.81**	**0.71–0.91**	**0.87**	**0.76–0.98**	**1.28**	**1.11–1.47**	**1.30**	**1.13–1.50**	**–**	**–**	**–**	**–**
- quite a bit/extremely	**0.85**	**0.73–0.98**	0.93	0.80–1.09	1.16	0.99–1.36	**1.21**	**1.02–1.44**	**–**	**–**	**–**	**–**
Self-Rated Overall Health												
- excellent/very good (ref)	1.00	–	1.00	–	1.00	**–**	1.00	**–**	**–**	**–**	**–**	**–**
- good	**1.28**	**1.14–1.44**	1.10	0.97–1.24	**0.79**	**0.68–0.91**	0.89	0.76–1.03	**–**	**–**	**–**	**–**
- fair/poor	**2.23**	**1.94–2.56**	**1.51**	**1.27–1.79**	**0.49**	**0.41–0.58**	**0.71**	**0.58–0.87**	**–**	**–**	**–**	**–**

Abbreviations: *OR* odds ratio; *CI* confidence interval; *ref* reference category; *MIZ* metropolitan influenced zone; *BMI* body mass index; *HUI* health utility index

^a^Province of residence included in all final adjusted models. Bolded values are statistically significant at *P* < 0.05 level. Only variables that were retained in the final models for each dependent variable are reported in the table. Only variables that were retained in the final models for each dependent variable are reported in the table

The following summarizes the multivariate analysis findings with only variables that were significant in the final models listed. Factors associated with use of a family physician ‘only’ were: older age (i.e. > 34 years); female; lower educational attainment; lower income; immigrant status; current smoker; having comorbidities other than CBD; less pain limiting function; less self-rated stress; lower overall self-rated health. Factors associated with ‘any’ use of a chiropractor included: higher educational attainment; higher income; Caucasian (versus non-Caucasian); non-smoker; having less than 3 co-morbidities; moderate pain-limited function; higher self-rated stress; better overall self-rated health. Factors associated with ‘any’ use of a physiotherapist included: younger age (< 50 years); female; higher educational attainment; higher income; urban residence; other ethnicity (versus Caucasian); non-smoker; more physically active; more pain-limited function. The vast majority of the odds ratios presented in the final adjusted models are statistically significant (Table 3). Despite the statistical significance of the results, most of the associations are of moderate strength (OR = 1.4–1.9 or their complement as an inverse association, 0.71–0.50). A few of the odds ratios are above 2 (< 0.5) (e.g. older age, higher income quintiles, number of co-morbidities), which is generally thought to be considered a stronger association in epidemiologic studies.

## Discussion

The majority of adult respondents with CBD sought care only with a family physician (53.8%), with fewer respondents seeking care with the following combination of providers: chiropractor/family physician (20.9%); physiotherapist/family physician (16.2%); physiotherapist/chiropractor (0.3%); family physician/chiropractor/physiotherapist (5.0%). Few respondents sought care only with a chiropractor (2.5%) or physiotherapist (1.0%). After adjustment, differential patterns of utilization (*p* < 0.05) among those with CBD were evident between provider groups. Characteristics of adults with CBD who reported no use of particular care providers included: older adults (any physiotherapist use); men (any physiotherapist use and only family physician use); lower educational attainment (any physiotherapist use and any chiropractor use); lower income (any physiotherapist use and any chiropractor use); Aboriginal or other non-Caucasian ethnicity (any chiropractor use); rural residence (any physiotherapist use); smokers (any chiropractor use and any physiotherapist use); 3 or more co-morbidities (any chiropractor use); and lower physical activity levels (any chiropractor use and any physiotherapist use).

Prior research investigating health care use among adult Canadians with CBD using the 2000–2001 CCHS [4] found some similar patterns to the present study, with some exceptions which may arise from different methodological approaches and data availability. Notably, the prior work did not use the restricted data files for the CCHS available through Statistics Canada, thus was limited in the type and level of variables that could be investigated (e.g. residence not included, income was only a dichotomous variable). Also, no multivariate analysis was performed to control for possible confounding. For example, we found an income gradient of greater use of physiotherapy and chiropractor services among those with progressively higher incomes and an inverse relationship of income levels with family physician usage. These findings regarding socioeconomic status are similar to research comparing family physician and chiropractic service usage in Saskatchewan [22] and Toronto, Ontario [23]. The vast majority of chiropractors and approximately half of physiotherapy providers in Canada work in the private sector [24], thus being able to access these services for CBD will be largely dependent on having additional health insurance, which an estimated one third of Canadians do not have [25]. Paradoxically, possessing private health insurance for uninsured health costs is highly associated with income [26], yet Canadians with lower incomes are more likely to have CBD [2]. Similarly, rural residents of Canada are nearly 30% more likely than urban dwellers to experience CBD [2], with limited access to appropriate health care proposed as a contributing factor to higher prevalence rates [27–29]. Poor access to publicly funded physiotherapy services and lack of multidisciplinary team support in rural settings have been identified as issues by Canadian physicians [30]. We found lower use of physiotherapy services among rural and remote respondents with no difference between rural and urban respondents in use of either family physician or chiropractor services; this is counter to what others have found in relation to chiropractic services [22]. Our findings may reflect people with CBD who either are more likely to travel to receive physician or chiropractic services in urban centres, or increased service delivery and/or providers in rural and remote settings.

A recently published ‘call to action’ on the global problem of low back pain highlighted the risks of overmedicalization of low back pain with excessive medical investigations and low value health care approaches that increase the risk of long-term back-related disability [31]. Ensuring equitable access to affordable health care for those who need it was among the several recommendations arising from this expert panel. The relatively high proportion of respondents in this study who reported seeking care ‘only’ with a family physician (fully publicly funded within the Canada) suggests that many people with CBD are receiving a predominantly medical approach to care. Enhancing access to non-pharmacological treatment options, such as physiotherapy or chiropractic care, for people with CBD is an important public health issue in Canada, particularly in light of the current opiod crisis. The first and last recommendations of the 2017 Canadian Guideline for Opiods for Chronic Non-cancer Pain [32] include referral to multidisciplinary non-physician care providers; however, given the access barriers to these services, these guidelines become challenging to follow in many clinical settings. Several models of care that include physiotherapists or chiropractors within primary care settings have shown benefit [33–36], and others that are attempting to overcome access barriers are being investigated [37, 38].

This study is one of the few known analyses that provides a national picture of self-reported health care use among people with CBD that accounts for multiple socio-demographic, and health characteristics; however, there are several limitations to consider. The main limitation is that due to the design of the CCHS, we were unable to determine the reason *why* respondents sought care. Although only half of those who experience low back pain are thought to consult a health care provider, most of those who consult have experienced symptoms for a longer period of time [39]. Thus our focus on CBD would take into account those with more prolonged symptoms who may be more likely to seek care. To focus on community-based health care provider use, our analysis did not include people who were hospitalized in the past year. However, this likely removed some respondents who also sought care with health care providers outside of the hospital. We did not include ‘any’ use family physician services in this analysis in order to focus predominantly on ‘only’ use of predominantly publically funded services. The small sample sizes of physiotherapy or chiropractor service use ‘only’ precluded us from profiling these as mutually exclusive groups. As such, there is overlap in participants who reported seeking care with each of these provider groups. The usage patterns of people with CBD (Fig. 1); however, suggests that, aside from family physicians, multiple provider usage is more common than seeking care with a single provider. Our dependent variable was a minimum of a single visit in the past year. Examination of patterns of use with multiple visits over time may yield different results. This study is based on data that is several years old; however, our as of yet unpublished analysis of newer survey data (i.e. 2014 for physiotherapist use only among general population) suggests similar gaps in access among rural and lower income populations. Furthermore, we are not aware of any significant policy or funding changes that have been implemented in Canada that would suggest the current patters of use would be substantially different. Self-reported health care utilization is not likely to be as accurate as administrative data; however, there is no available administrative data source in Canada to comparatively examine these health care provider groups. Finally, the cross-sectional design does not permit conclusions regarding the direction of the observed associations.

## Conclusions

This research highlights potential inequities in access to physiotherapists and chiropractors in relation to family physicians among adult Canadians with CBD, particularly among lower socioeconomic status and rural/remote populations. The identified gaps in access to care among certain population groups demonstrates that there is not equitable access to care among Canadians with CBD. Enhancing access to potentially beneficial non-physician services for people with CBD requires rethinking the way front-line back care is delivered in Canada, including pressure on insurers and policy makers to cover and enable greater access to non-pharmacological management treatment options that have demonstrated value [34, 40].

## Abbreviations

BMI: Body Mass Index
CBD: chronic back disorders
CCHS: Canadian Community Health Survey
CI: confidence interval
HUI: Health Utility Index
MIZ: Metropolitan Influenced Zone
OR: Odds ratio
